# Evidence of Ringtail Expansion Into Idaho, USA

**DOI:** 10.1002/ece3.73477

**Published:** 2026-04-28

**Authors:** Peter F. Rebholz, Robert C. Lonsinger, David E. Ausband

**Affiliations:** ^1^ Idaho Cooperative Fish and Wildlife Research Unit University of Idaho Moscow Idaho USA; ^2^ U.S. Geological Survey, Oklahoma Cooperative Fish and Wildlife Research Unit Oklahoma State University Stillwater Oklahoma USA; ^3^ U.S. Geological Survey, Idaho Cooperative Fish and Wildlife Research Unit University of Idaho Moscow Idaho USA

## Abstract

Ringtails ( *Bassariscus astutus* ) are widely distributed in the western United States and across much of Mexico, yet due to their relatively low densities and cryptic nature, little is known about their basic ecology even where they are common. Recent public sightings suggest ringtails may have expanded their distribution north into southern Idaho, USA. Currently, ringtails remain unclassified in Idaho thus limiting resources and time available for population monitoring. We attempted to detect these small carnivores in southern Idaho during winter 2023. We deployed 49 camera traps with a combination of lures (i.e., trapping lure, fruit, and orange drink mix) in canyon habitats in southern Idaho near recent ringtail sightings. We detected ringtails nine times on six different camera traps. Although incidental ringtail observations increased in southern Idaho since the early 2000s, our detections were the first to result from a targeted survey effort. Given more than 20 years of incidental sightings and multiple detections during our survey, it appears that ringtails have expanded their range northward into southern Idaho and could be considered a resident species in the state. Further efforts to document reproduction, annual persistence, and an assessment of threats could be next steps if the state wildlife management agency deems it a priority and an appropriate use of resources.

## Graphical abstract

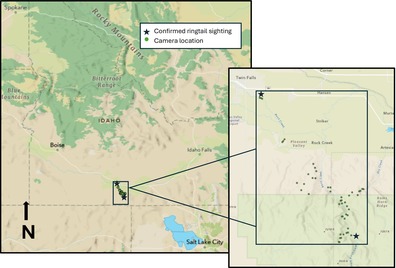

## 1 Introduction

Ringtails ( *Bassariscus astutus* ) are a widely occurring nocturnal procyonid, but little is known about their ecology even where they are common (Gundermann et al. 2023). Ringtails can be found across most of Mexico and the southwestern United States; their distribution in the United States ranges north along the west coast to Oregon and east to Arkansas (Poglayen‐Neuwall and Toweill 1988; Gehrt 2003). Ringtails are typically solitary, except during their breeding season between the end of February and May (Toweill 1976; Poglayen‐Neuwall and Toweill 1988).

Ringtails are adaptable habitat generalists that can occupy a variety of habitats including those with human disturbance (Poglayen‐Neuwall and Toweill 1988). The ringtail is omnivorous and typically consumes small mammals (e.g., rodents), fruit, birds, and insects (Trapp 1978; Rodríguez‐Estrella et al. 2000). Ringtail habitat characteristics and dietary tendencies vary geographically (Poglayen‐Neuwall and Toweill 1988; Rodríguez‐Estrella et al. 2000; Ackerson and Harveson 2006). The land‐cover type that ringtails occupy varies greatly and includes oak (*Quercus* spp.) and temperate rainforests, grasslands, piñon (*Pinus* spp.) and juniper (*Juniperus* spp.) woodlands, and scrublands, but they are most often associated with landscapes having canyons and rocky vertical relief (Poglayen‐Neuwall and Toweill 1988; Gundermann et al. 2023). Although early research asserted that ringtails required access to free‐standing water (Cahalane 1954; Taylor 1954), Richards (1976) and Chevalier (1984) showed that ringtails have kidneys adapted to conserving water and can survive in arid conditions. Ringtail habitat selection may relate to access to diurnal resting and denning sites to shelter them from weather, predators, and for space to raise young (Dalke 1948; Callas 1987); resting and denning sites are opportunistically occupied by ringtails and are primarily found in rock and tree cavities (Toweill 1976).

Despite the relatively broad distribution of ringtails in western North America, few studies have been conducted on ringtail ecology and habitat use, with even fewer studies in low‐density populations near the species' northern range extent (Ackerson and Harveson 2006; Harrison 2012; Gundermann et al. 2023). The northernmost extent of the ringtail distribution is southwestern Oregon (Lonsinger and Roemer 2024), but ringtails have been documented infrequently in eastern Oregon (Bailey 1936), southern Idaho (Larrison 1967), and southwestern Wyoming (Long and House 1961). Investigating the occurrence and habitat use of species at their range edge—where environmental quality is likely reduced for the species relative to the core of their range—can provide insight into factors limiting the species' distribution (Geber 2008).

Permanent occupation by ringtails in southern Idaho has not been substantiated, but increases in the number of ringtail observations suggest there may be a resident population in southern Idaho. In 2003, there was a verified ringtail carcass found in southern Idaho near Castle Rocks State Park (Wildlife Health Laboratory 2003). Additional observations of ringtail tracks (2005) and a sighting (2006) were reported in City of Rocks National Reserve (Vincent et al. 2007). Three ringtails have been captured in anthropogenic environments of southern Idaho and relocated by wildlife managers in 2014 (Liebenthal 2014), 2015 (Hutchins 2015), and 2021 (Thompson 2022). A single scientific survey for ringtails has been conducted in southern Idaho (within City of Rocks National Reserve and Castle Rocks State Park); this survey sampled 30 sites over 1260 camera trap nights during the springs of 2011 and 2012, but failed to detect the species (Lonsinger 2012). To date, ringtails have not been considered a resident species within Idaho's State Wildlife Action Plan (IDFG 2024). Ringtails have a State Conservation Rank indicating the species is “nonbreeding” and “accidental [or] casual” within the state.

Mesopredator populations have been expanding in recent decades, perhaps due to loss of apex predators (i.e., mesopredator release: Crooks and Soulé 1999; Prugh et al. 2009). Additionally, land‐cover changes such as woody tree encroachment from fire suppression and temperature changes may facilitate colonization in new areas, particularly along northern edges of a species' existing range. Documenting range expansions can be difficult for low‐density carnivores that are elusive and challenging to observe directly. Fortunately, camera traps allow us to remotely identify animals and survey a variety of locations 24 h/day (Burton et al. 2015). Because of recent public sightings, we wanted to determine if ringtails were expanding their distribution north into Idaho, potentially from extant populations in Utah, Nevada, or southern Wyoming. Thus, we sought to detect ringtails in southern Idaho near recent sightings during their breeding season using camera traps.

## 2 Methods

### 2.1 Study Area

**FIGURE 1:**
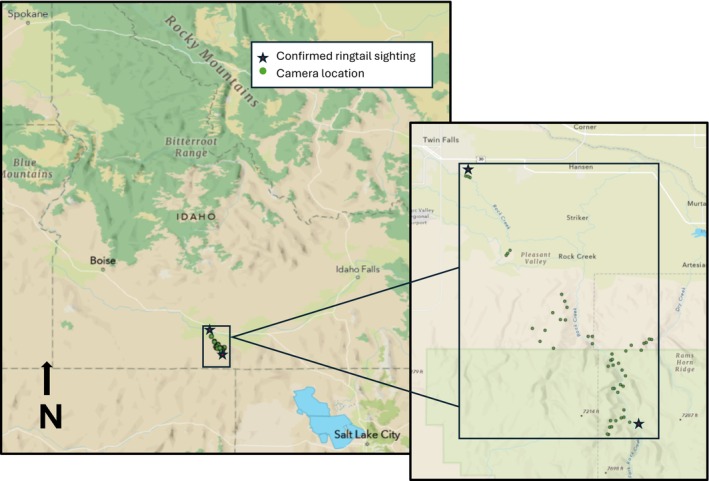
Camera locations (indicated by green circle) for a 2022–2023 ringtail survey in the Rock Creek canyon system in Southern Idaho, USA (Esri 2025). Recently confirmed ringtail sightings by Idaho Fish and Game (indicated by red star).

We deployed baited camera stations on public and private lands in and around the Sawtooth National Forest south of Twin Falls, ID, USA. Our study area occurred within the Rock Creek canyon system near the most recent confirmed ringtail sighting (Figure 1). Portions of the Sawtooth National Forest within our study area hosted sagebrush (*Artemisia* spp.) and juniper that blended into aspen (*Populus* spp.), lodgepole pine ( *Pinus contorta* ), and fir (*Abies* spp.) at higher elevations. Land ownership in the area was a mix of public and private properties and there was extensive urban and suburban development within the area including commercial, livestock grazing, industrial, and residential areas. During our surveys, the study area experienced typical temperatures ranging from a February mean of −8°C, the coldest month, to an April mean of 1°C, the warmest month (PRISM Climate Group 2025). The southern hills of south‐central Idaho receive a mean annual precipitation of 61 cm between February and April (PRISM Climate Group 2025).

### 2.2 Field Methods

We deployed cameras at 49 non‐randomized sites between February and April 2023 (Figure 1). We deployed one Reconyx PC900 (Holmen, WI) camera at each site and recorded independent species detections. We deployed each camera at a height of 1.0–3.0 m, oriented cameras toward game trails, and programmed cameras to have high sensitivity, use a covert infrared flash, no delay/quiet period, and take a 3‐image burst per trigger. Cameras were deployed in the canyon and tributary drainages along Rock Creek between the most recent confirmed ringtail sightings (Figure 1). We used satellite imagery to find a variety of canyonland cover types (e.g., riparian, grassland, rim‐rock, and juniper woodlands); we then hiked in each of these habitats and located active game trails for camera deployment. Camera locations varied in elevation (mean = 1558 m, SD = 175.5) with lower elevation sites in the northern half of the study area near residential and commercial properties, whereas higher elevation sites were more remote and farther from anthropogenic features. The majority of sites were set within the higher elevation and more remote southern half of the study area (*n* = 44), whereas fewer sites were set at lower elevations near anthropogenic features in the north (*n* = 6). At each camera site, we applied an olfactory attractant on a prominent object (e.g., large rock, log, or bone). Additionally, we soaked a tampon with a lure, which we hung ~3.0 m high in trees between 2.0 and 6.0 m in front of the camera. For our olfactory attractant, we used a combination of bait (banana, strawberry jam; ~60 mL), long‐distance lures (~0.5 mL per site; Mega Musk, Carman Lures; Long Distance Call, O'Gorman Enterprises, Broadus, MT), and Tang soft drink powder (~30 mL). We then manually viewed each image taken from all 49 cameras, summed the number of ringtail detections for each camera, and recorded date, time, and location of each ringtail detection. We recorded a detection event as a “unique detection” of a ringtail when a camera was triggered 24 h after the last ringtail triggering event.

## 3 Results

**FIGURE 2:**
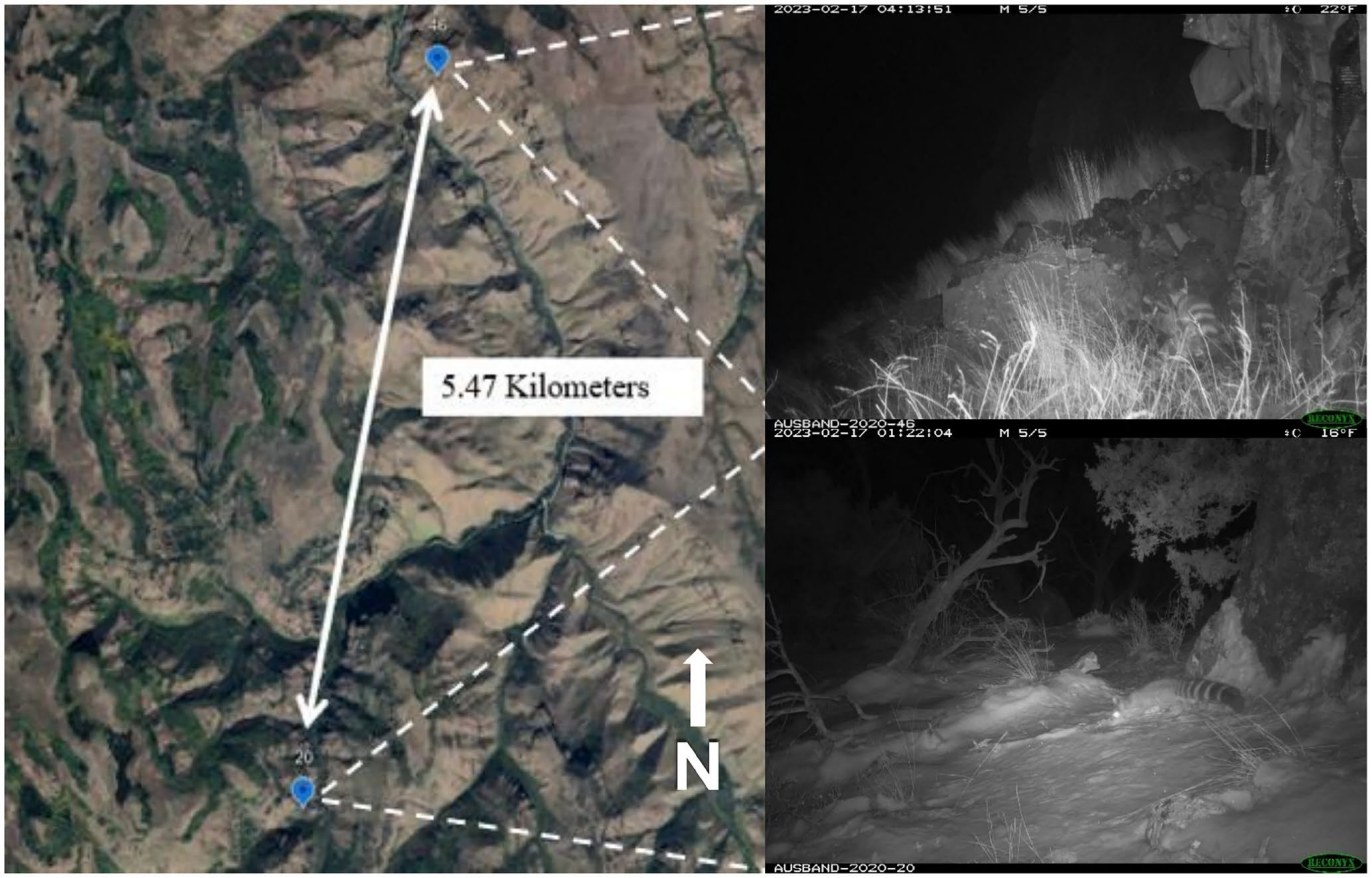
Locations of two cameras (#46 and #20) 5.47 km apart (linearly) and separated by Rock Creek, which captured the presence of ringtails on 17 February 2023 within 3 h of one another in southern Idaho, USA.

We recorded 54 images of ringtails from nine unique detections across six different cameras between 20 Feb 2023 and 8 May 2023 (*n* = 82, average number of trap nights). Five detections (83%) were at sites characterized by riparian habitat lined with trees and elevations between 1463 and 1829 m (Table 1), whereas one detection (16%) was at a site located near a water seep in a mix of grassland and rimrock habitat. All detections were at night or early morning between 20:00 and 6:00, with 7 of 9 unique detections (78%) occurring on different days. Mean latency to detection for ringtails was 36 trap nights (SD = 29.03, range = 5–83). We detected ringtails on two different cameras on 17 Feb 2023 within 3 h of each other (Figure 2); these cameras were > 5.4 km apart (linearly) and separated by Rock Creek, suggesting these detections were of two different individual ringtails (Figure 2).

**TABLE 1:** Results from camera trap survey targeting ringtails in southern Idaho, USA, 2023. Cameras #23, #20, #46, #30, #63 were set in riparian habitat while camera #33 was set in rimrock habitat.

Camera ID	Date (2023) and time of detection	Lat	Long	Number of trap nights to ringtail detection	Elevation (m)	Distance to open water (m)
#23	10 Feb—20:34	42.253	−114.252	5	1573	7
#23	25 Feb—05:59	42.253	−114.252	21	1573	7
#20	17 Feb—01:22	42.234	−114.270	14	1829	60
#33	12 Mar—01:01	42.249	−114.267	27	1737	120
#33	19 Mar—19:31	42.249	−114.267	34	1737	120
#33	8 May—03:51	42.249	−114.267	83	1737	120
#46	17 Feb—04:13	42.281	−114.258	5	1676	6
#30	18 Apr—04:26	42.287	−114.262	63	1463	5
#63	24 Apr—04:02	42.286	−114.265	69	1570	65
Mean (SD)	—	—	—	36 (29)	1558 (175)	56 (56)

## 4 Discussion

We documented ringtail occurrence in southern Idaho during the species' breeding season (February–May), suggesting that ringtails may have expanded their distribution and may now be resident in the area. Although we were unable to identify unique ringtails through camera‐based sampling, our detection patterns suggested there were likely multiple unique ringtails detected. Notably, home range size and characteristics can vary substantially among land‐cover types and reported mean home range sizes for ringtails have ranged from < 0.5 km^2^ to > 5 km^2^ (Lonsinger and Roemer 2024), and we detected ringtails on two cameras separated by > 5.4 km and a significant body of water within 3 h of each other. Our nine ringtail detections spanned 3 months, suggesting detections were unlikely representative of transient individuals and that ringtails have established in Idaho. Given more than 20 years of incidental sightings and multiple detections during our survey, it appears that ringtails have expanded their range northward into southern Idaho and could be considered a resident species in the state. Further efforts to document reproduction, annual persistence, and an assessment of threats could be next steps if the state wildlife management agency deems it a priority and an appropriate use of resources.

Over the last 20 years, there have been several carnivore and mesocarnivore camera‐based studies conducted in this region of southern Idaho, including City of Rocks and the canyon systems southwest of Twin Falls, Idaho, USA (Lonsinger 2012). These studies failed to detect ringtails in southern Idaho. It is possible that changing environmental factors such as juniper expansion may be facilitating ringtail range expansion into Idaho from established populations in Utah and Nevada (Monaco and Gunnell 2020). The expansion of juniper may provide additional vertical structure on the landscape for escape terrain, denning habitat, and a stable food source from the juniper berries. Research has shown that juniper berries in other populations are a prevalent food source in ringtail diets (Harrison 2012).

Due to the gap in knowledge of ringtails in their northmost range, it is unclear if and how changing habitat and environmental factors might affect these populations. In Oregon, USA, their farthest range northwest, Gundermann et al.'s (2023) study showed that northern populations of ringtails frequently select tree cavities as rest sites, and the availability of these microhabitats may be vital for the species' persistence. Oregon's state wildlife agency has determined that these habitat‐driven data gaps in ringtail abundance, distribution, and habitat selection qualify them for Species of Greatest Conservation Need status under the state's Wildlife Action Plan (ODFW 2026).

Ringtails are typically viewed as habitat generalists, but there is evidence from other portions of the species' range that individual preferences for particular habitat features could contribute to nonrandom dispersal (Lonsinger et al. 2015). We still do not know much about their movements, particularly in areas with low densities. Although we did not have sufficient data to formally assess the role of water availability or habitat structure on patterns of ringtail occurrence, we were only successful in detecting ringtails at cameras deployed within < 0.16 km from an open water source and 83% of our detections were in areas with large live trees. Southern Idaho's canyon system offers a suite of resource opportunities for an adaptable species such as ringtails. Ringtails are extremely variable in diet and den site selection. If managers seek to understand the distribution of ringtails in Idaho and have the resources to do so, our results indicate that cameras are an effective monitoring approach that could be deployed in a more extensive and robust sampling design. Additionally, concurrent genetic sampling could help answer questions about the genetic health and potential source population of southern Idaho's ringtails (Kleeberg et al. 2025).

## Author Contributions

**Peter F. Rebholz:** conceptualization (equal), data curation (lead), formal analysis (lead), investigation (lead), methodology (equal), project administration (equal), software (equal), supervision (equal), validation (lead), visualization (equal), writing – original draft (lead), writing – review and editing (lead). **Robert C. Lonsinger:** conceptualization (equal), data curation (equal), investigation (equal), methodology (equal), resources (equal), validation (equal), writing – original draft (equal), writing – review and editing (equal). **David E. Ausband:** conceptualization (equal), data curation (equal), formal analysis (equal), funding acquisition (equal), investigation (equal), methodology (equal), project administration (lead), resources (equal), software (equal), supervision (lead), validation (equal), visualization (equal), writing – original draft (equal), writing – review and editing (equal).

## Funding

This work was supported by Summerlee Foundation.

## Conflicts of Interest

The authors declare no conflicts of interest.

## Data Availability

All data are available in the table and figure in this manuscript.
